# A Free Web-Based Protocol to Assist Structure-Based Virtual Screening Experiments

**DOI:** 10.3390/ijms20184648

**Published:** 2019-09-19

**Authors:** Nathalie Lagarde, Elodie Goldwaser, Tania Pencheva, Dessislava Jereva, Ilza Pajeva, Julien Rey, Pierre Tuffery, Bruno O. Villoutreix, Maria A. Miteva

**Affiliations:** 1 Laboratoire GBCM, EA7528, Conservatoire National des Arts et Métiers, 2 Rue Conté, Hésam Université, 75003 Paris, France; nathalie.lagarde@lecnam.net; 2 Inserm U1268 MCTR, CNRS UMR 8038 CiTCoM - Univ. de Paris, Faculté de Pharmacie de Paris, 4 av de l’Observatoire, CEDEX 06, 75270 Paris, France; elodie.goldwaser@inserm.fr; 3 Department of QSAR and Molecular Modelling, Institute of Biophysics and Biomedical Engineering, Bulgarian Academy of Sciences, 105 Acad G. Bonchev Str., 1113 Sofia, Bulgaria; tania.pencheva@biomed.bas.bg (T.P.); dessi@biomed.bas.bg (D.J.); pajeva@biomed.bas.bg (I.P.); 4 Université de Paris, BFA, UMR 8251, CNRS, ERL U1133, Inserm, RPBS, F-75013 Paris, France; julien.rey@univ-paris-diderot.fr (J.R.); pierre.tuffery@univ-paris-diderot.fr (P.T.); 5 University of Lille, Inserm, Institut Pasteur de Lille, U1177, F-59000 Lille, France; bruno.villoutreix@inserm.fr

**Keywords:** web server, virtual screening, docking, molecular mechanics, structure refinement, ADME-Tox

## Abstract

Chemical biology and drug discovery are complex and costly processes. In silico screening approaches play a key role in the identification and optimization of original bioactive molecules and increase the performance of modern chemical biology and drug discovery endeavors. Here, we describe a free web-based protocol dedicated to small-molecule virtual screening that includes three major steps: ADME-Tox filtering (via the web service FAF-Drugs4), docking-based virtual screening (via the web service MTiOpenScreen), and molecular mechanics optimization (via the web service AMMOS2 [Automatic Molecular Mechanics Optimization for in silico Screening]). The online tools FAF-Drugs4, MTiOpenScreen, and AMMOS2 are implemented in the freely accessible RPBS (Ressource Parisienne en Bioinformatique Structurale) platform. The proposed protocol allows users to screen thousands of small molecules and to download the top 1500 docked molecules that can be further processed online. Users can then decide to purchase a small list of compounds for in vitro validation. To demonstrate the potential of this online-based protocol, we performed virtual screening experiments of 4574 approved drugs against three cancer targets. The results were analyzed in the light of published drugs that have already been repositioned on these targets. We show that our protocol is able to identify active drugs within the top-ranked compounds. The web-based protocol is user-friendly and can successfully guide the identification of new promising molecules for chemical biology and drug discovery purposes.

## 1. Introduction

Chemical biology and drug discovery are complex and costly processes and usually involve high-throughput screening campaigns, computations, and/or wet lab experiments, prioritization of the hit compounds, and different levels of compound optimization. In silico screening methodologies play a key role in the identification and optimization of original bioactive molecules and increase the performance of modern chemical biology and drug discovery endeavors. [1,2,3,4,5,6]. These approaches can assist the various stages of drug discovery and have impressively progressed during the last decades. Still, several challenges exist, such as how to deal with the flexibility of the binding pocket [7], how to improve scoring [8], and how to automate the processes among others [9,10,11]. To date, several web services have been developed in that direction: de novo drug design (e-LEAD3 [12]), docking of several small molecules (e.g., SwissDock [13], CovalentDock [14]), and predicting binding affinities of protein–ligand complexes [15]. Some other services are more specialized in large-scale virtual ligand screening (e.g., iScreen [16], DOCK Blaster [17], USR-VS [18]).

**Figure 1:**
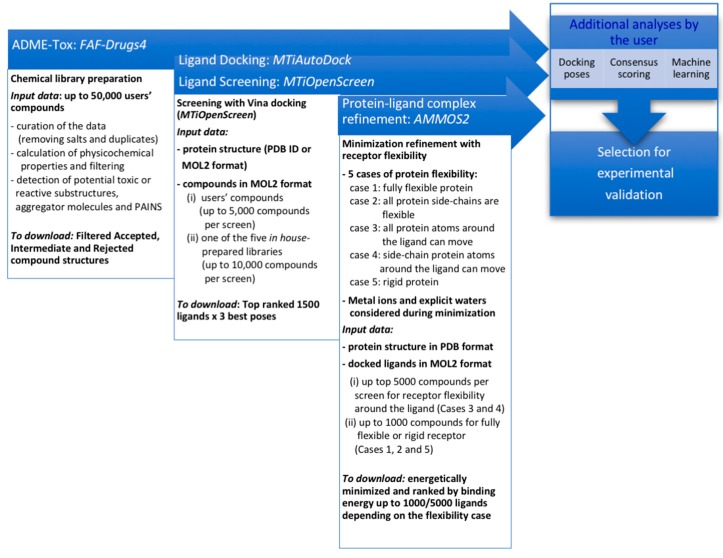
A general workflow of the main steps in the virtual screening protocol employing the three web servers: FAF-Drugs4, MTiOpenScreen, and AMMOS2. The web servers can be used independently or successively depending on the particular needs of the user.

Here, we describe a free web-based protocol (Figure 1) dedicated to small-molecule virtual screening that includes three major steps and combines three previously reported web servers (Figure 1): ADME-Tox filtering via the web server FAF-Drugs4 [19] (http://fafdrugs4.mti.univ-paris-diderot.fr), docking-based virtual screening via the web server MTiOpenScreen [20] (http://bioserv.rpbs.univ-paris-diderot.fr/services/MTiOpenScreen/), and molecular mechanics optimization to refine the docked complexes via the web server AMMOS2 (Automatic Molecular Mechanics Optimization for in silico Screening) [21] (http://drugmod.rpbs.univ-paris-diderot.fr/ammosHome.php). The three web servers are implemented in the RPBS (Ressource Parisienne en Bioinformatique Structurale) [22], a platform dedicated to structural bioinformatics computations (about 60,000 connections per year). The RPBS computer system controls job execution, storage, resource quota, etc. The data are private for each user. RPBS jobs are submitted to a 1000-core cluster running on CentOS operating system and managed by the Slurm workload manager. The performance of each of the three web servers has already been thoroughly evaluated [19,20,21]. To demonstrate the potential of an online-based protocol, including a successive application of the three web servers, we performed virtual screening experiments of 4574 approved drugs against three cancer targets (CDK2, GP130, and cereblon), and the results were analyzed in the light of drugs reported to be repositioned for these targets.

## 2. Results and Discussion

### 2.1. A Free Web-Based Protocol Dedicated to Small-Molecule Virtual Screening

#### 2.1.1. Chemical Compound Preparation

The first step of our protocol (Figure 1) is the preparation of a chemical library with physicochemical properties appropriate for the user’s screening project. For that purpose, one can use FAF-Drugs4, which is a free web-based package that allows compound libraries to be filtered based on physicochemical rules, undesirable toxic/reactive groups, and pan-assay interference compounds (PAINS) [23]. The first version of the service was released in 2006 [24]. The user-friendly interface of FAF-Drugs4 facilitates the analysis of the compound library of up to 50,000 small molecules online. The major functionalities in FAF-Drugs4 include the following: a data curation procedure that encompasses search for salts, physicochemical parameter filtering, solubility prediction, prediction of blood–brain barrier penetration, computations of the Pfizer 3/75 (preclinical toxicity) [25] and of the GSK 4/400 (high risk of toxicity) [26] rules, search for toxicophores, detection of putative inhibitors of protein–protein interactions, and drug-induced phospholipidosis prediction [27]. In addition, the Eli Lilly open drug discovery medicinal chemistry filter for open drug discovery [28] is implemented. The user can generate the desired chemical library using various options of FAF-Drugs4 for subsequent analysis or virtual screening. FAF-Drug4 generates filtered chemical libraries containing molecules protonated at physiological pH using the pKa values calculated by the Chemaxon software (www.chemaxon.com). Finally, the molecules filtered by FAF-Drugs4 can be piped to the RPBS web server Frog2 for the generation of single or multiple 3D conformations [29] (http://bioserv.rpbs.univ-paris-diderot.fr/services/Frog2). FAF-Drugs4 was used to prepare the chemical library Drug-lib screened in this work (see Methods section and Table S1 for details). FAF-Drugs4 took 25 min to filter the merged 8394 drug structures for the preparation of the Drugs-lib library. On average, FAF-Drugs4 takes 3 h to filter a chemical library of 50, 000 compounds [19].

#### 2.1.2. Docking-Based Virtual Screening

The filtered chemical compound collection prepared with FAF-Drugs4 can be uploaded in the MTiOpenScren web server. The MTiOpenScreen web server performs docking and virtual screening of small molecules, offering the possibility to screen in one run up to 5000 molecules uploaded by the user or up to 10,000 molecules taken from the 170,000 compounds ready to dock provided by RPBS. Two services—MTiAutoDock and MtiOpenScreen—are available. MTiAutoDock, based on AutoDock 4.2 [30], performs docking into a binding pocket defined by the user or blind docking over the entire protein surface. The blind docking with MTiAutoDock takes, on average, 25 min for a protein receptor requiring a grid of 170 × 170 × 170 points for a grid spacing of 0.6 Å [20]. For the three proteins screened here (CDK2, GP130, and cereblon), the bind docking of the corresponding drug took, on average, 10 min. MTiOpenScreen based on AutoDock Vina docking [31] performs automated virtual ligand screening. MTiOpenScreen provides original valuable starting collections for screening. One can screen up to 10,000 compounds from five in-house prepared libraries containing drug-like molecules. Users can apply physicochemical filters accessible in MTiOpenScreen to further select the molecules for their projects. The Diverse-library (Diverse-lib) and the library of molecules likely inhibiting protein–protein interaction (iPPI-lib) contain 99,288 and 51,232 drug-like molecules, respectively. In addition, MTiOpenScreen provides screening of purchasable approved drugs (Drugs-lib containing 7173 stereoisomers corresponding to 4574 single isomer molecules), food (FOOD-lib containing 10,997 stereoisomers corresponding to 3015 single isomer molecules), and natural (NP-lib containing 1228 stereoisomers corresponding to 653 single isomer molecules) compound collections [32]. Thus, MTiOpenScreen ensures virtual screening experiments on diverse chemical libraries for classical protein targets or protein–protein interactions [33,34,35]. One can screen up to 10,000 compounds in a binding site of dimensions 25 × 25 × 25 Å in 1 h [20], keeping in mind that RPBS can treat ~170 MTiOpenScreen queries per week. As such, it can take more time in some situations depending on the server loads. The screening of Drugs-lib on three proteins (CDK2, GP130, and cereblon) (see Methods section and Table S1 for details) took, on average, 1.5 h per protein.

#### 2.1.3. Molecular Mechanics Refinement

AMMOS2 is a web server [21] that executes automatic energy minimization of experimental or docked protein–ligand complexes at an atomic-level using the molecular mechanics modeling program AMMP [36] and the AMMOS software [37]. AMMOS2 allows minimization of a large number of ligands at different levels of flexibility of the protein receptor, allowing moving of the following: all atoms of the protein (case 1, a fully flexible protein); all atoms of the protein side chains (case 2); all protein atoms inside a sphere around the bound ligand (case 3); all protein atoms of the protein side chains inside a sphere around the bound ligand (case 4); and none of the protein atoms (case 5, a rigid protein). Up to 1000 ligands are accepted as input for cases 1, 2, and 5. For cases 3 and 4, the limit is up to 5000 ligands. The radius of the sphere around the bound ligand is of user’s choice, with values ranging from 4 Å to 8 Å for cases 3 and 4. In addition, AMMOS2 considers explicit water molecules and metal ions belonging to the protein receptor during minimization. The user can download the structures of the minimized protein–ligand complexes as well as the predicted protein–ligand binding energies and the ligand ranks according to the minimized binding energies. AMMOS2 also ensures interactive analysis of the 100 top-ranked ligands, thus favoring users to collect data for further studies. We performed minimization with AMMOS2 and the flexibility cases 1, 3, and 4 for the docked protein–ligand complexes generated by MTiOpenScreen on three proteins (CDK2, GP130, and cereblon) (see Methods section and Table S1 for details). AMMOS2 took, on average, 8 min per protein for the minimization of 1000 ligand poses and 45 min per protein for minimization of 4500 ligand poses.

### 2.2. Screening of Approved Drugs Using the Web-Based Protocol for Drug Repositioning

To evaluate the performance of our web-based protocol for virtual screening (see Table S1), we chose three cancer targets for which repositioned drugs have been reported. The chemical library Drugs-lib, containing 7173 stereoisomers corresponding to 4574 single isomer molecules available at the MTiOpenScreen service and screened here, was previously prepared with the FAF-Drugs4 web server (see Methods section for details). We screened the Drugs-lib collection against three cancer targets using MTiOpenScreen and analyzed the performance of our protocol to identify the known repositioned drugs within the top 1500 scores. The docked protein–ligand complexes were finally optimized and re-ranked with AMMOS2.

#### 2.2.1. Fluspirilene

**Figure 2:**
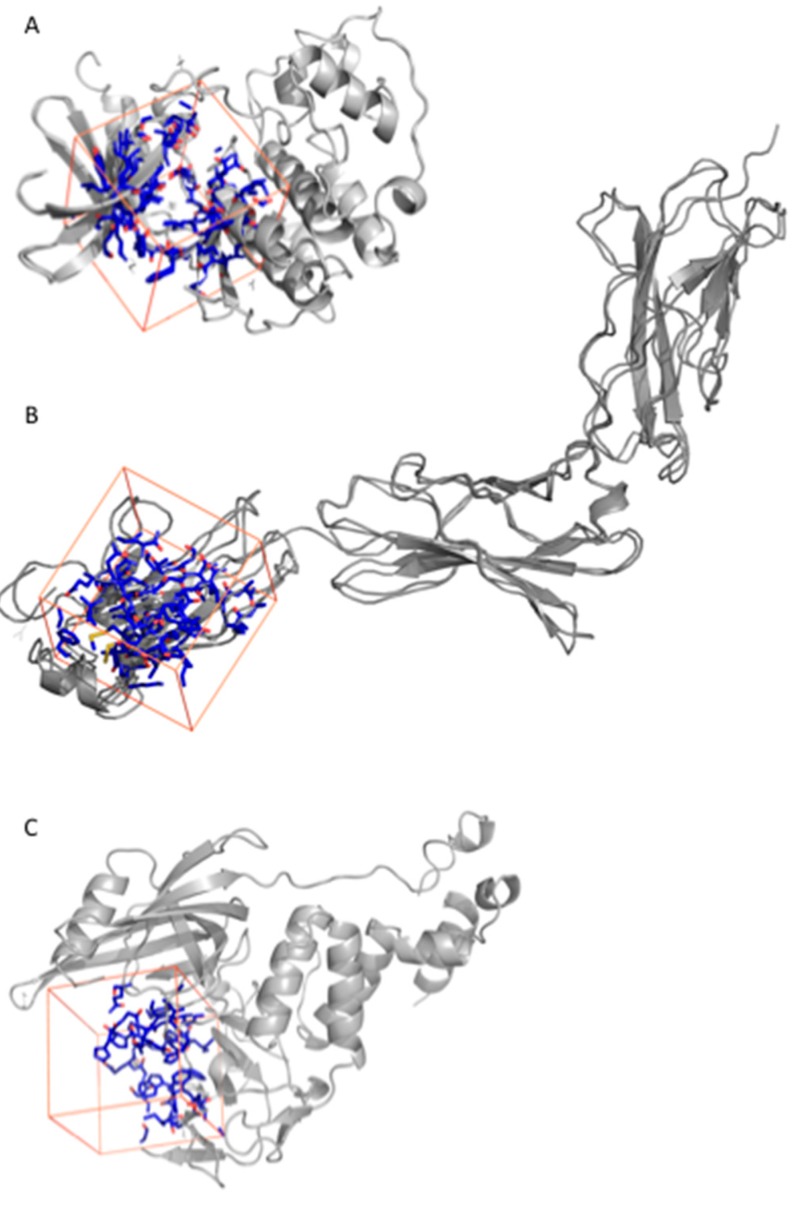
Visual representation of the AutoDock Vina box search space (in orange) for (**A**) CDK2 (Protein Data Bank (PDB) IDs: 1PXI and 1VYZ), (**B**) GP130 (PDB IDs: 1P9M and 3L5H), and (**C**) cereblon (PDB ID: 4CI1). All structures used for this study are superimposed and represented in gray cartoon; the residues of the binding sites are represented in blue sticks.

**Figure 3:**
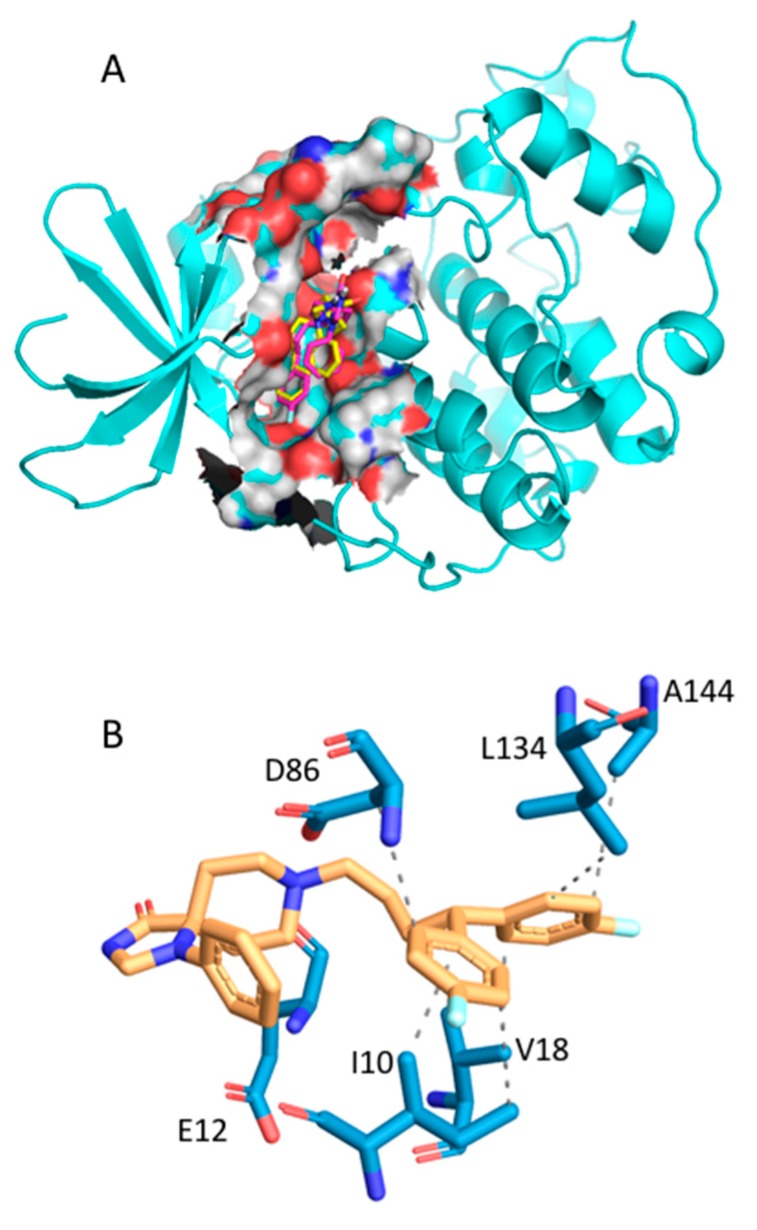
(**A**) Best-scoring fluspirilene binding poses as docked into CDK2 (PDB ID: 1VYZ) by MTiOpenScreen’s Vina (in yellow atom-type sticks) and as minimized by AMMOS2 case 1 (in magenta atom-type sticks). The CDK2 structure is shown in cartoon, and the ATP binding site is shown as spheres. (**B**) PLIP image as generated by the AMMOS2 web server for the fluspirilene binding pose (in yellow atom-type sticks) as minimized by AMMOS2 case 1. The protein residues interacting with the fluspirilene are shown in blue atom-type sticks.

Fluspirilene [38] is an antipsychotic drug used for therapy of schizophrenia patients. Fluspirilene is known to inhibit the dopamine D2 receptor [39] and to block a calcium channel [40]. In 2015, Shi et al. [41] reported virtual ligand screening of ~4900 US Food and Drug Administration (FDA)-approved small-molecule drugs that allowed finding of CDK2 as a new target for fluspirilene. In vitro and in vivo experiments confirmed that fluspirilene could be used as a new anticancer drug for hepatocellular carcinoma treatment. CDK2 is a cyclin-dependent kinase involved in cell replication and tumor growth, and it is a promising cancer target [42]. A large number of CDK2 inhibitors have been published. Huge structural information is available for CDK2 as 358 human CDK2 structures cocrystallized with a ligand (holo structures) are deposed in the Protein Data Bank (PDB). In our previous study [32], 44 CDK2 structures were used to probe fluspirilene as an inhibitor of CDK2 [41]. For those structures, except 1E1V, fluspirilene was found in the top 1500 best-ranked compounds, with ranks from 83^rd^ to 1048^th^ position. The AutoDock Vina scores were calculated to be from −12.2 to −8.4 kcal/mol. In order to probe the capability of AMMOS2 molecular mechanics optimization to improve the ranking of fluspirilene after virtual screening computations with MTiOpenScreen, we used two PDB structures—1PXI and 1VYZ (see Figure 2A)—on which MTiOpenScreen did not show the best performance [32]. Fluspirilene was ranked by MTiOpenScreen at positions 805 and 443, with calculated AutoDock Vina scores equal to −8.7 and −9.2 kcal/mol for the PDB 1PXI and 1VYZ, respectively (Table 1). Three AMMOS2 runs were executed for cases 1, 3, and 4. For 1PXI, the three AMMOS2 cases improved the rank to 520, 665, and 654, respectively. For 1VYZ, only case 1 minimization improved the rank to 342 (Figure 3A). AMMOS2 minimization improved the binding energies, considering electrostatic and van der Waals interactions, for all protein–ligand complexes. Very high protein–ligand interaction energies were computed by AMMOS2 prior to minimization as clashes were present after Vina docking because nonpolar atoms cannot be optimized during Vina docking. Both MTiOpenScreen and case 1 AMMOS2 minimization identified fluspirilene as active in the top 10% on the screened Drugs-lib library when using the PDB structure 1VYZ. The specific interactions between the fluspirilene and CDK2 as optimized by AMMOS2 case 1 are shown in Figure 3B.

#### 2.2.2. Raloxifene

**Figure 4:**
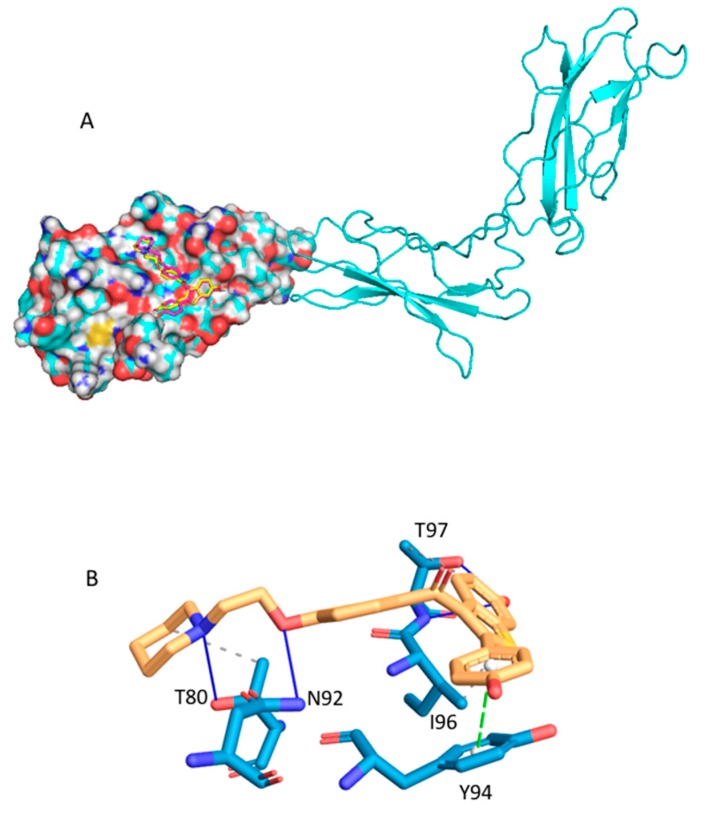
(**A**) Best-scoring raloxifene binding poses as docked into GP130 (PDB ID: 1P9M) by MTiOpenScreen’s Vina (in yellow atom-type sticks) and as minimized by AMMOS2 case 1 (in magenta atom-type sticks). The GP130 domain D1, where the raloxifene is predicted to bind, is shown as spheres. Domains D2 and D3 are shown in cartoon. (**B**) PLIP image as generated by the AMMOS2 web server for the raloxifene binding pose (in yellow atom-type sticks) as minimized by AMMOS2 case 1. The protein residues interacting with the raloxifene are shown in blue atom-type sticks.

Raloxifene is a well-known nonhormonal drug used for the prevention and therapy of postmenopausal osteoporosis [43]. Raloxifene is a selective estrogen receptor modulator, which binds to two estrogen receptors (ER): ER_alpha and ER_beta [44]. Recently, raloxifene and its analogue bazedoxifene were identified as new inhibitors of the PPI interaction IL-6(Interleukin-6)/GP130 using an in silico protocol [45]. IL-6 and GP130 participate in the key IL-6/JAK/STAT3 pathway, which is involved in proliferation and metastasis of tumor cells. The IL-6/JAK/STAT3 pathway is also involved in the suppression of the anticancer immune response and is thus a promising cancer target [46]. Subsequent in vitro experiments confirmed the potential use of raloxifene and its analogues for IL-6/GP130/STAT3-dependent cancer treatment. Li et al. [45] proposed that the binding site of raloxifene is in the GP130 protein, at the interface between the GP130 and the IL-6 D1 domain. Three crystal structures of the GP130 protein with present D1 domain are found in the PDB (PDB IDs: 1I1R, 1P9M, 3L5H). MTiOpenScreen was used to dock the Drugs-lib collection into the 1P9M structure (Figure 2). The raloxifene score was computed to be −6.5 kcal/mol (Table 1) with a rank of 737 (Figure 4A). We then reproduced the same protocol using the PDB structure 3L5H. When using 3L5H, raloxifene rank was 126, and the computed MTiOpenScreen score was −7.5 kcal/mol. Three AMMOS2 runs were subsequently executed for 1P9M and 3L5H for cases 1, 3, and 4. For 1P9M, the three AMMOS2 cases improved the rank to 292, 455, and 466, respectively. MTiOpenScreen and case 1 AMMOS2 minimization identified raloxifene as active in the top 10% on the screened Drugs-lib library when using the PDB structures 3L5H and 1P9M, respectively. The specific interactions between the raloxifene and GP130 as optimized by AMMOS2 case 1 are shown in Figure 4B. For 3L5H, AMMOS2 minimization could not improve the rank of raloxifene.

#### 2.2.3. Thalidomide

**Figure 5:**
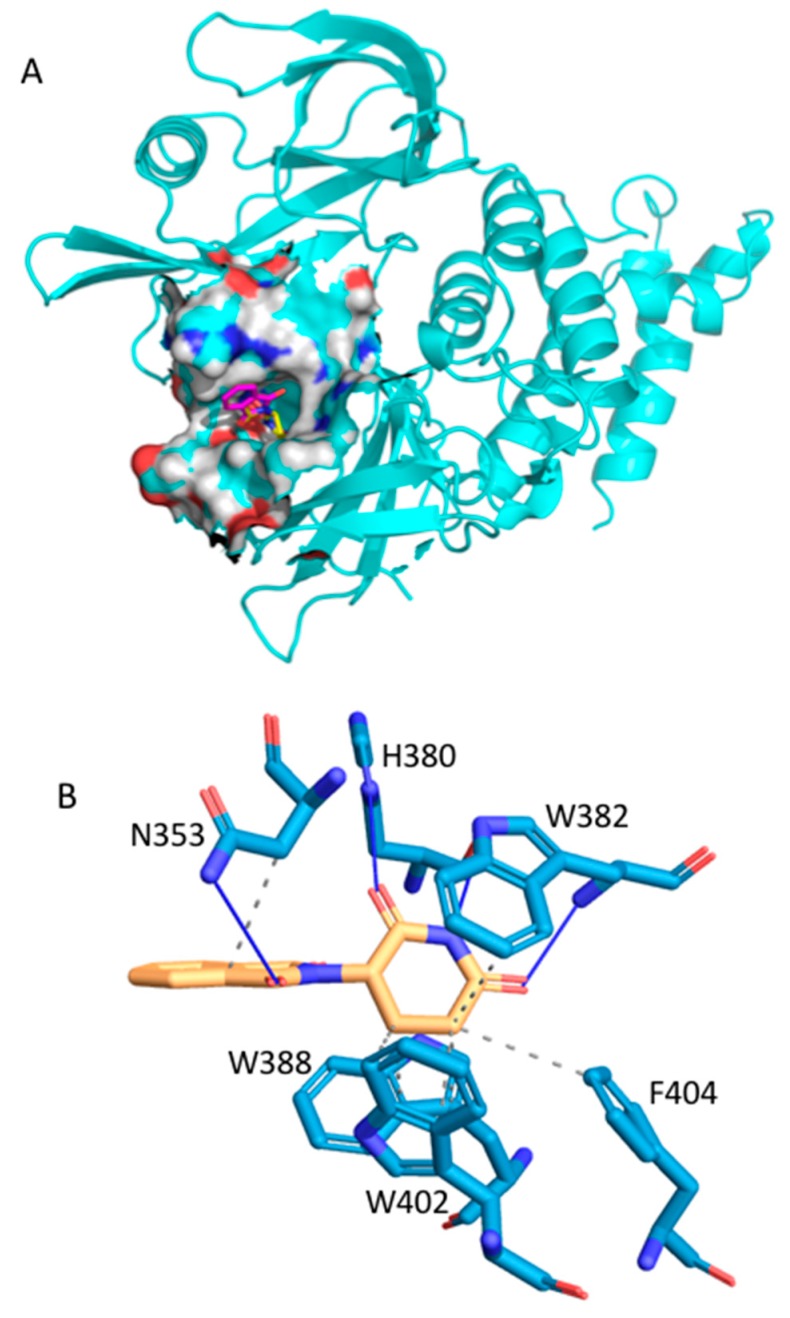
(**A**) Best-scoring thalidomide binding poses as docked into cereblon (PDB ID: 4CI1) by MTiOpenScreen’s Vina (in yellow atom-type sticks) and as minimized by AMMOS2 case 1 (in magenta atom-type sticks). The cereblon structure is shown in cartoon, and the thalidomide binding site is shown as spheres. (**B**) PLIP image as generated by the AMMOS2 web server for the thalidomide binding pose (in yellow atom-type sticks) as minimized by AMMOS2 case 1. The protein residues interacting with the thalidomide are shown in blue atom-type sticks.

Thalidomide is another well-known example of drug repositioning. It was marketed in 1956 as a sedative also used to prevent morning sickness in pregnancy and withdrawn in 1963 after discovery of severe teratogenic effects presented by babies exposed to thalidomide in utero [47]. However, thalidomide has demonstrated a wide range of immunomodulatory and antiangiogenic effects. It has been successfully used for multiple myeloma treatment [48] as activator of cereblon [49], which is a part of the E3 ubiquitin ligase complex. Two transcription factors involved in B cell development (IKZF1 and IKZF3) highly expressed in multiple myeloma are downregulated by this activation. Seven holo crystal structures of human cereblon are available in the PDB. MTiOpenScreen was used to dock the Drugs-lib library into the PDB ID structure 4CI1 (Figure 2). The thalidomide score was computed to be −9.8 kcal/mol (Table 1) with a rank of 196. Three AMMOS2 runs were then executed for flexibility cases 1, 3, and 4. The ligand poses obtained by Vina docking and the poses obtained by case 1 AMMOS2 minimization were almost identical (Figure 5A), in accordance with their similar binding energies calculated by AMMOS2 before and after minimization. Case 1, with the obtained rank of 341, performed the best among the three AMMOS2 minimizations. Yet, in this case, the minimization could not improve the rank of thalidomide achieved by MTiOpenScreen, probably due to a near-optimal positioning of the compound by Vina and the associated high score. Both MTiOpenScreen and case 1 AMMOS2 minimization identified thalidomide as active in the top 10% of the screened Drugs-lib library. The specific interactions between the compound and cereblon after AMMOS2 case 1 computations are shown in Figure 5B.

## 3. Materials and Methods

### 3.1. Web Servers

#### 3.1.1. FAF-Drugs4 Web Server

The FAF-Drugs4 software includes seven object-oriented Python modules. The molecules considered by Faf-Drugs4 are presented as a molecular object using the OpenBabel toolkit [50]. The major molecule treatment proposed by FAF-Drugs4 includes the following: (i) curation (removing large molecules, some inorganic atoms, salts, and duplicates); (ii) computation of physicochemical characteristics; (iii) detection of potential toxic or reactive substructures, aggregator molecules, and PAINS (137 substructure alerts and 515 PAINS); and (iv) result reports and downloading. Users can design their compound library using several predefined physicochemical filters: a Lipinski’s rule of five filter [51], a RO3 filter for fragments [52], a probe-like filter [53], the REOS filter [54], the ZINC drug-like filter [55], a CNS filter [56] based on molecules known to pass through the blood–brain barrier, and a respiratory filter [57] based on inhaled or intranasal-administered drugs. In-house developed drug-like and lead-like filters are also available, which are based on the physicochemical characteristics of 916 oral FDA-approved drugs [58]. Advanced users can use their own project-dependent physicochemical ranges. The filtered compounds are divided into three files: Accepted.sdf (according to the used physicochemical filter without any structural alerts found), Intermediate.sdf (low-risk structural alerts found), Rejected.sdf (molecules that do not pass the chosen physicochemical filter or high-risk structural alert identified), and PAINS.sdf.

Input: The FAF-Drugs4 web server accepts only SDF files. The service Bank-Formatter accessible via the RPBS web portal (http://mobyle.rpbs.univ-paris-diderot.fr/cgi-bin/portal.py?form=FAF-Drugs4#forms::Bank-Formatter) prepares the input file into a suitable SDF file for FAF-Drugs4 if needed and also uses as input molecules in SMILES format. Output: The following data can be downloaded: the filtered compound files Accepted.sdf, Intermediate.sdf, Rejected.sdf, and PAINS.sdf; results.csv (the physicochemical properties), groups.csv (the structural alert searches found), and pains.csv (PAINS compounds found). More information on each compound can be obtained by opening another web-based page (e.g., indicating detected problems, a PCA (principal component analysis) comparing the selected compound with the oral drug chemical space, radar plots of the physicochemical properties, and toxic subgroups identified.

#### 3.1.2. MTiOpenScreen Web Server

MTiAutoDock performs blind docking on the entire protein surface or in a user-defined binding site for up to 10 ligands using AutoDock4.2 [30]. For MTiAutoDock, the maximum grid dimensions are set to 200 × 200 × 200 with resolution of 0.375 Å. In cases where this resolution is not sufficient to cover the entire protein surface, the grid resolution can change to 0.6 or 0.8 Å. The screening service MTiOpenScreen [20] is based on the program AutoDock Vina [31] to execute docking for virtual screening experiments. AutoDock Vina employs empirical scoring and a gradient-based conformational docking. The parameters used in MTiOpenScreen service are as follows: grid resolution is 1 Å, the number of output poses is 10, and the exhaustiveness level is 8. For the binding pocket definition, one can provide a list of residues or upload the grid dimensions and center data.

Input: Users should provide the protein structure in MOL2 or PDB format. For virtual screening with MTiOpenScreen, users can upload a compound library that should not exceed 5000 molecules. Output: MTiOpenScreen provides an interactive page [59] showing the 3D protein structure and the 100 top-ranked ligands. The structures and the binding energies of the 1500 top-ranked ligands can be downloaded. Users can perform additional analysis of the best putative ligands using free software like PyMOL (www.pymol.org/) or AutoDockTools [30] or can pipeline the downloaded docked poses to AMMOS2 for pose optimization and rescoring.

#### 3.1.3. AMMOS2 Web Server

AMMOS2 allows molecular mechanics optimization of protein–ligand complexes applying the sp4 AMMP force field developed on the basis of the UFF potential set [60] and the AMBER partial charges [61]. A fast multipole algorithm is employed for the nonbonded energy terms without the use of a cutoff radius, allowing to speed the computations [36]. For the minimization procedure, AMMOS2 employs conjugate gradient optimization (2 × 500 iterations). All optimized ligands are ranked by the final receptor–ligand binding energy [37].

Input: The protein receptor should be in PDB format, and the bound ligands should be in MOL2 format. Five levels of protein flexibility are permitted. Missing hydrogen atoms of the ligands can be added by AMMOS2, which is practical when the ligand docking positions are generated by MTiOpenScreen. Output: AMMOS2 returns to user the structures of the minimized ligands ranked by the binding energies, the optimized protein–ligand complexes structures, the computed energies, the images, and the analysis report generated by the PLIP software [62]. The PLIP report displays hydrogen bonds, hydrophobic interactions, cation–pi, and pi–pi interactions. Additionally, PLIP software generates an image of the predicted interactions in the binding pocket. For the 100 top-ranked ligands, the 3D structures of the minimized complexes are visualized by the AMMOS2 web service.

### 3.2. Preparation of Data for Performance Assessment

#### 3.2.1. Compound Collection Preparation

Four compound databases were used to generate the library Drugs-lib: the “drug” subset of the ChEMBL database [63]; the “approved” subset of DrugBank [64]; the DrugCentral database [65]; and the “approved” SuperDrug2 database [66]. We used FAF-Drugs4 [19] to remove isotopes, inorganics, mixtures, salts, and duplicates. We employed in-house soft filter for physicochemical properties in order to keep compounds with molecular weight of 100 to 1000 and <20 rotatable bonds. Then, we removed compounds with detected toxicophores and PAINS A, B, and C [67]. Finally, we selected the “Accepted” and “Intermediate” molecules. We kept only purchasable compounds (following the ZINC15 database [68]). The compounds were protonated at pH 7 using ChemAxon [69]. CORINA Classic [70] was used for the 3D conformation generation to preserve the existing stereocenters.

#### 3.2.2. Protein Structure Preparation and Calculation Parameters

For MTiOpenScreen, the protein structure can be directly taken from the PDB or prepared using the widely used free package Chimera for protonation and alignment as done here [71].

Fluspirilene: Here, we used two PDB structures of CDK2, PDB IDs: 1PXI and 1VYZ. The two proteins were aligned. For the MTiOpenScreen Vina docking, we used grid center coordinates of 12.036, 46.084, and 24.851 (using 1PXI as reference) and a grid size of 20 Å × 20 Å × 20 Å.

Raloxifene: Here, we used two PDB structures of the human GP130 proteins, which include the GP130-IL6 interface domain, PDB IDs: 1P9M and 3L5H. For the MTiOpenScreen Vina docking, we used grid center coordinates of −101.693, 216.308, and 44.304 (using 1P9M as reference) and a grid size of 20 Å × 20 Å × 20 Å.

Thalidomide: The PDB ID structure 4CI1 of cereblon was used here. For the MTiOpenScreen Vina docking, we used grid center coordinates of −1.227, 3.848, and 12.907 (using 4CI1 as reference) and a grid size of 20 Å × 20 Å × 20 Å.

AMMOS2 minimization runs were executed for case 1 (full flexible protein and ligand), case 3 (all atoms of the protein inside a sphere of 6 Å around the ligand and the ligand are flexible), and case 4 (all atoms of the protein side chains inside a sphere of 6 Å around the ligand and the ligand are flexible).

## 4. Conclusions

The web-based protocol using three free web servers allows users with no training in computer sciences to perform virtual ligand screening for chemical biology or drug discovery projects. The preparation of the chemical library is of critical importance for the success of the virtual screening exercise. FAF-Drug4 can filter a chemical library of up to 50,000 small molecules using curation, calculation of key physicochemical properties, and identification of potential toxic and PAINS molecules. MTiOpenScreen carries out virtual screening via the implemented docking program AutoDock Vina. One can screen up to 5000 user-uploaded molecules or 10,000 small molecules present in the various in-house prepared chemical libraries (diverse drug-like compounds, approved drugs, food compounds, natural product compounds, molecules dedicated to inhibit protein–protein interactions). The last stage proposed here involves molecular mechanics optimization by AMMOS2 of the top 1000 or 1500 protein–ligand complexes generated by MTiOpenScreen. The performance tests of the web-based protocol executed here on three cancer targets showed that the three repositioned drugs were systematically found by MTiOpenScreen and cases 1, 3, and 4 AMMOS2 minimization in the 1000 top-ranked compounds. In fact, for each target, the repositioned drug was found by MTiOpenScreen and case 1 AMMOS2 minimization in the top 10% of the screened library for at least one protein structure. The differences in ranking obtained for the different protein structures suggest that it could be valuable to employ “ensemble docking” approaches (e.g., multiple conformations of the receptor). Further, in most cases, the final AMMOS2 minimization with flexible protein receptor (AMMOS2 case 1) improved the drug ranking. Overall, AMMOS2 cases 3 and 4, which allow flexibility of the binding site, showed similar results, with slightly better performance for case 3. Yet, AMMOS2 did not systematically improve the rank obtained by MTiOpenScreen for all examined protein structures. Thus, in practice, it will be critical to consider the rank lists obtained by MTiOpenScreen and by AMMOS2. If it is not possible to employ AMMOS2 case 1, which is limited to 1000 molecules, case 3, which accepts up to 5000 molecules and shows sound results, is an appropriate solution. More experienced users can perform postprocessing, such as rescoring, taking into account the docked protein–ligand complex structures as optimized by the AMMOS2 minimization protocol. Final visual inspection of the top-ranked compounds is in general recommended in order to privilege the best and diverse candidates for the subsequent experimental validation.

## Figures and Tables

**Table 1:** MTiOpenScreen and AMMOS2 screening for three proteins.

Drug, PDB ID	AutoDock Vina		AMMOS2 Case 1			AMMOS2 Case 3			AMMOS2 Case 4		
	score	rank	Energy before min	Energy after min	rank	Energy before min	Energy after min	rank	Energy before min	Energy after min	rank
1	2	3	4	5	6	7	8	9	10	11	12
Fluspirilene 1PXI	−8.7	805	207.75	3.98	520	195.32	8.96	665	>1000.0	15.97	654
Fluspirilene 1VYZ	−9.2	443	111.96	−29.37	342	657.01	−13.31	683	>1000.0	−8.51	619
Raloxifene 3L5H	−7.5	126	>1000.0	−72.11	480	>1000.0	−59.01	609	>1000.0	−25.50	850
Raloxifene 1P9M	−6.5	737	−49.74	−124.65	292	−49.73	−119.48	455	−49.74	−114.09	466
Thalidomide 4CI1	−9.8	196	−38.11	−40.31	341	−16.34	−41.49	578	−38.11	−40.31	555

The first column indicates the PDB ID of the protein X-ray structure used and the corresponding drug. The second and third columns report on the score and the rank of the corresponding drug attributed by AutoDock Vina. The fourth, fifth, and sixth columns present the protein–ligand interaction energies before and after AMMOS2 case 1 minimization and the corresponding rank. The seventh, eighth, and ninth columns present the protein–ligand interaction energies before and after AMMOS2 case 3 minimization and the corresponding rank. The last three columns present the protein–ligand interaction energies before and after AMMOS2 case 4 minimization and the corresponding rank. The interaction energies calculated by AMMOS2 include electrostatic and van der Waals interactions between the ligand and the protein atoms included in the minimization. All ranks are based on the best single isomer among 4574 drugs of the Drugs-lib library.

## Supplementary Materials

Supplementary Materials can be found at https://www.mdpi.com/1422-0067/20/18/4648/s1.

## Abbreviations

RPBS	Ressource Parisienne en Bioinformatique Structurale
AMMOS2	Automatic Molecular Mechanics Optimization for in silico Screening
PAINS	pan-assay interference compounds
CDK2	cyclin-dependent kinase 2
ER	estrogen receptor
FDA	food drug administration
IL-6	interleukin-6
MW	molecular weight
PDB	Protein Data Bank

## References

[1] Jabeen A., Ranganathan S. (2019). Applications of machine learning in GPCR bioactive ligand discovery. Curr. Opin. Struct. Biol..

[2] Cavasotto C.N., Orry A.J. (2007). Ligand Docking and Structure-based Virtual Screening in Drug Discovery. Curr. Top. Med. Chem..

[3] Ma D.L., Chan D.S., Leung C.H. (2013). Drug repositioning by structure-based virtual screening. Chem. Soc. Rev..

[4] Kar S., Roy K. (2013). How far can virtual screening take us in drug discovery?. Expert Opin. Drug Discov..

[5] Gautier B., Miteva M.A., Goncalves V., Huguenot F., Coric P., Bouaziz S., Seijo B., Gaucher J.F., Broutin I., Garbay C. (2011). Targeting the proangiogenic VEGF-VEGFR protein-protein interface with drug-like compounds by in silico and in vitro screening. Chem. Biol..

[6] Chevillard F., Lagorce D., Reynes C., Villoutreix B.O., Vayer P., Miteva M.A. (2012). In silico prediction of aqueous solubility: A multimodel protocol based on chemical similarity. Mol. Pharm..

[7] Moroy G., Sperandio O., Rielland S., Khemka S., Druart K., Goyal D., Perahia D., Miteva M.A. (2015). Sampling of conformational ensemble for virtual screening using molecular dynamics simulations and normal mode analysis. Future Med. Chem..

[8] Li H., Peng J., Sidorov P., Leung Y., Leung K.S., Wong M.H., Lu G., Ballester P.J. (2019). Classical scoring functions for docking are unable to exploit large volumes of structural and interaction data. Bioinformatics.

[9] Scior T., Bender A., Tresadern G., Medina-Franco J.L., Martinez-Mayorga K., Langer T., Cuanalo-Contreras K., Agrafiotis D.K. (2012). Recognizing pitfalls in virtual screening: A critical review. J. Chem. Inf. Modeling.

[10] Ain Q.U., Aleksandrova A., Roessler F.D., Ballester P.J. (2015). Machine-learning scoring functions to improve structure-based binding affinity prediction and virtual screening. Wiley Interdiscip. Rev. Comput. Mol. Sci..

[11] Yuriev E., Holien J., Ramsland P.A. (2015). Improvements, trends, and new ideas in molecular docking: 2012–2013 in review. J. Mol. Recognit..

[12] Douguet D. (2010). e-LEA3D: A computational-aided drug design web server. Nucleic Acids Res..

[13] Grosdidier A., Zoete V., Michielin O. (2011). SwissDock, a protein-small molecule docking web service based on EADock DSS. Nucleic Acids Res..

[14] Ouyang X., Zhou S., Ge Z., Li R., Kwoh C.K. (2013). CovalentDock Cloud: A web server for automated covalent docking. Nucleic Acids Res..

[15] Pires D.E., Ascher D.B. (2016). CSM-lig: A web server for assessing and comparing protein-small molecule affinities. Nucleic Acids Res..

[16] Tsai T.Y., Chang K.W., Chen C.Y. (2011). iScreen: World’s first cloud-computing web server for virtual screening and de novo drug design based on TCM database@Taiwan. J. Comput.-Aided Mol. Des..

[17] Irwin J.J., Shoichet B.K., Mysinger M.M., Huang N., Colizzi F., Wassam P., Cao Y. (2009). Automated docking screens: A feasibility study. J. Med. Chem..

[18] Li H., Leung K.S., Wong M.H., Ballester P.J. (2016). USR-VS: A web server for large-scale prospective virtual screening using ultrafast shape recognition techniques. Nucleic Acids Res..

[19] Lagorce D., Bouslama L., Becot J., Miteva M.A., Villoutreix B.O. (2017). FAF-Drugs4: Free ADME-tox filtering computations for chemical biology and early stages drug discovery. Bioinform. (Oxf. Engl.).

[20] Labbe C.M., Rey J., Lagorce D., Vavrusa M., Becot J., Sperandio O., Villoutreix B.O., Tuffery P., Miteva M.A. (2015). MTiOpenScreen: A web server for structure-based virtual screening. Nucleic Acids Res..

[21] Labbe C.M., Pencheva T., Jereva D., Desvillechabrol D., Becot J., Villoutreix B.O., Pajeva I., Miteva M.A. (2017). AMMOS2: A web server for protein-ligand-water complexes refinement via molecular mechanics. Nucleic Acids Res..

[22] Alland C., Moreews F., Boens D., Carpentier M., Chiusa S., Lonquety M., Renault N., Wong Y., Cantalloube H., Chomilier J. (2005). RPBS: A web resource for structural bioinformatics. Nucleic Acids Res..

[23] Baell J.B., Holloway G.A. (2010). New substructure filters for removal of pan assay interference compounds (PAINS) from screening libraries and for their exclusion in bioassays. J. Med. Chem..

[24] Miteva M.A., Violas S., Montes M., Gomez D., Tuffery P., Villoutreix B.O. (2006). FAF-Drugs: Free ADME/tox filtering of compound collections. Nucleic Acids Res..

[25] Hughes J.D., Blagg J., Price D.A., Bailey S., Decrescenzo G.A., Devraj R.V., Ellsworth E., Fobian Y.M., Gibbs M.E., Gilles R.W. (2008). Physiochemical drug properties associated with in vivo toxicological outcomes. Bioorg. Med. Chem. Lett..

[26] Gleeson M.P. (2008). Generation of a set of simple, interpretable ADMET rules of thumb. J. Med. Chem..

[27] Przybylak K.R., Alzahrani A.R., Cronin M.T. (2014). How does the quality of phospholipidosis data influence the predictivity of structural alerts?. J. Chem. Inf. Modeling.

[28] Bruns R.F., Watson I.A. (2012). Rules for identifying potentially reactive or promiscuous compounds. J. Med. Chem..

[29] Miteva M.A., Guyon F., Tuffery P. (2010). Frog2: Efficient 3D conformation ensemble generator for small compounds. Nucleic Acids Res..

[30] Morris G.M., Huey R., Lindstrom W., Sanner M.F., Belew R.K., Goodsell D.S., Olson A.J. (2009). AutoDock4 and AutoDockTools4: Automated docking with selective receptor flexibility. J. Comput. Chem..

[31] Trott O., Olson A.J. (2010). AutoDock Vina: Improving the speed and accuracy of docking with a new scoring function, efficient optimization, and multithreading. J. Comput. Chem..

[32] Lagarde N., Rey J., Gyulkhandanyan A., Tuffery P., Miteva M.A., Villoutreix B.O. (2018). Online structure-based screening of purchasable approved drugs and natural compounds: Retrospective examples of drug repositioning on cancer targets. Oncotarget.

[33] Mullard A. (2012). Protein-protein interaction inhibitors get into the groove. Nat. Rev. Drug Discov..

[34] Zhang X., Betzi S., Morelli X., Roche P. (2014). Focused chemical libraries--design and enrichment: An example of protein-protein interaction chemical space. Future Med. Chem..

[35] Villoutreix B.O., Kuenemann M.A., Poyet J.L., Bruzzoni-Giovanelli H., Labbe C., Lagorce D., Sperandio O., Miteva M.A. (2014). Drug-like protein-protein interaction modulators: Challenges and opportunities for drug discovery and chemical biology. Mol. Inform..

[36] Weber I.T., Harrison R.W. (1997). Molecular mechanics calculations on Rous sarcoma virus protease with peptide substrates. Protein Sci.: A Publ. Protein Soc..

[37] Pencheva T., Lagorce D., Pajeva I., Villoutreix B.O., Miteva M.A. (2008). AMMOS: Automated Molecular Mechanics Optimization tool for in silico Screening. BMC Bioinform..

[38] Janssen P.A., Niemegeers C.J., Schellekens K.H., Lenaerts F.M., Verbruggen F.J., Van Nueten J.M., Schaper W.K. (1970). The pharmacology of penfluridol (R 16341) a new potent and orally long-acting neuroleptic drug. Eur. J. Pharmacol..

[39] Hassel P. (1985). Experimental comparison of low doses of 1.5 mg fluspirilene and bromazepam in out-patients with psychovegetative disturbances. Pharmacopsychiatry.

[40] Wang S.J. (2002). Inhibition of glutamate release by fluspirilene in cerebrocortical nerve terminals (synaptosomes). Synapse.

[41] Shi X.N., Li H., Yao H., Liu X., Li L., Leung K.S., Kung H.F., Lu D., Wong M.H., Lin M.C. (2015). In Silico Identification and In Vitro and In Vivo Validation of Anti-Psychotic Drug Fluspirilene as a Potential CDK2 Inhibitor and a Candidate Anti-Cancer Drug. PLoS ONE.

[42] Asghar U., Witkiewicz A.K., Turner N.C., Knudsen E.S. (2015). The history and future of targeting cyclin-dependent kinases in cancer therapy. Nat. Rev. Drug Discov..

[43] Cranney A., Adachi J.D. (2005). Benefit-risk assessment of raloxifene in postmenopausal osteoporosis. Drug Saf..

[44] Bryant H.U. (2001). Mechanism of action and preclinical profile of raloxifene, a selective estrogen receptor modulation. Rev. Endocr. Metab. Disord..

[45] Li H., Xiao H., Lin L., Jou D., Kumari V., Lin J., Li C. (2014). Drug design targeting protein-protein interactions (PPIs) using multiple ligand simultaneous docking (MLSD) and drug repositioning: Discovery of raloxifene and bazedoxifene as novel inhibitors of IL-6/GP130 interface. J. Med. Chem..

[46] Johnson D.E., O’Keefe R.A., Grandis J.R. (2018). Targeting the IL-6/JAK/STAT3 signalling axis in cancer. Nat. Rev. Clin. Oncol..

[47] Vargesson N. (2015). Thalidomide-induced teratogenesis: History and mechanisms. Birth Defects Res. Part C Embryo Today Rev..

[48] Singhal S., Mehta J., Desikan R., Ayers D., Roberson P., Eddlemon P., Munshi N., Anaissie E., Wilson C., Dhodapkar M. (1999). Antitumor activity of thalidomide in refractory multiple myeloma. New Engl. J. Med..

[49] Stewart A.K. (2014). Medicine. How thalidomide works against cancer. Science.

[50] O’Boyle N.M., Banck M., James C.A., Morley C., Vandermeersch T., Hutchison G.R. (2011). Open Babel: An open chemical toolbox. J. Cheminformatics.

[51] Lipinski C.A., Lombardo F., Dominy B.W., Feeney P.J. (2001). Experimental and computational approaches to estimate solubility and permeability in drug discovery and development settings. Adv. Drug Deliv. Rev..

[52] Congreve M., Carr R., Murray C., Jhoti H. (2003). A ‘rule of three’ for fragment-based lead discovery?. Drug Discov. Today.

[53] Workman P., Collins I. (2010). Probing the probes: Fitness factors for small molecule tools. Chem. Biol..

[54] Charifson P.S., Walters W.P. (2002). Filtering databases and chemical libraries. Mol. Divers..

[55] Irwin J.J., Shoichet B.K. (2005). ZINC--a free database of commercially available compounds for virtual screening. J. Chem. Inf. Modeling.

[56] Jeffrey P., Summerfield S. (2010). Assessment of the blood-brain barrier in CNS drug discovery. Neurobiol. Dis..

[57] Ritchie T.J., Luscombe C.N., Macdonald S.J. (2009). Analysis of the calculated physicochemical properties of respiratory drugs: Can we design for inhaled drugs yet?. J. Chem. Inf. Modeling.

[58] Pihan E., Colliandre L., Guichou J.F., Douguet D. (2012). e-Drug3D: 3D structure collections dedicated to drug repurposing and fragment-based drug design. Bioinform. (Oxf. Engl.).

[59] Biasini M. (2015). Zenodo. pv: v1.8.1 (Version V1.8.1).

[60] Rappé A.K., Casewit C.J., Colwell K.S., Goddard III W.A., Skiff W.M. (1992). UFF, a full periodic table force field for molecular mechanics and molecular dynamics simulations. J. Am. Chem. Soc..

[61] Weiner S.J., Kollman P.A., Nguyen D.T., Case D. (1986). An all atom force field for simulations of proteins and nucleic acids. J. Comput. Chem..

[62] Salentin S., Schreiber S., Haupt V.J., Adasme M.F., Schroeder M. (2015). PLIP: Fully automated protein-ligand interaction profiler. Nucleic Acids Res..

[63] Gaulton A., Bellis L.J., Bento A.P., Chambers J., Davies M., Hersey A., Light Y., McGlinchey S., Michalovich D., Al-Lazikani B. (2012). ChEMBL: A large-scale bioactivity database for drug discovery. Nucleic Acids Res..

[64] Wishart D.S., Feunang Y.D., Guo A.C., Lo E.J., Marcu A., Grant J.R., Sajed T., Johnson D., Li C., Sayeeda Z. (2018). DrugBank 5.0: A major update to the DrugBank database for 2018. Nucleic Acids Res..

[65] Ursu O., Holmes J., Knockel J., Bologa C.G., Yang J.J., Mathias S.L., Nelson S.J., Oprea T.I. (2017). DrugCentral: Online drug compendium. Nucleic Acids Res..

[66] Siramshetty V.B., Eckert O.A., Gohlke B.O., Goede A., Chen Q., Devarakonda P., Preissner S., Preissner R. (2018). SuperDRUG2: A one stop resource for approved/marketed drugs. Nucleic Acids Res..

[67] Lagorce D., Sperandio O., Baell J.B., Miteva M.A., Villoutreix B.O. (2015). FAF-Drugs3: A web server for compound property calculation and chemical library design. Nucleic Acids Res..

[68] Sterling T., Irwin J.J. (2015). ZINC 15--Ligand Discovery for Everyone. J. Chem. Inf. Modeling.

[69] ChemAxon. Marvin Calculator Plugins version 17.23. www.chemaxon.com.

[70] 3D Structure Generator CORINA Classic, Molecular Networks GmbH, Nuremberg, Germany. www.mn-am.com.

[71] Pettersen E.F., Goddard T.D., Huang C.C., Couch G.S., Greenblatt D.M., Meng E.C., Ferrin T.E. (2004). UCSF Chimera--a visualization system for exploratory research and analysis. J. Comput. Chem..

