# Supplementary material for: A Free Web-Based Protocol to Assist Structure-Based Virtual Screening Experiments
Source: Int J Mol Sci. 2019 Sep 19;20(18):4648. doi: 10.3390/ijms20184648 (PMC6769597; doi:10.3390/ijms20184648)
Supplement: Supplementary file 1 [file ijms-20-04648-s001.pdf]

Supplementary Materials:

Table 1. Main steps of the proposed web-based virtual screening protocol. The docking/scoring phase was applied to 4574 approved drugs. The calculation time is averaged from the screening of the three cancer protein targets, CDK2, GP130 and cereblon, investigated here.

Step	Webserver	Input	Output	Calculation Time	Link to Web Server and Userguide	Ref
1	**FAF-Drugs4** Filtering of chemical library according to physicochemical properties and toxic/PAINS groups	Merging 8394 drug structures from: 1) ChEMBL “drug” 2) DrugBank “approved” 3) Drug Central 4) SuperDrug2 “approved”	Drugs-lib available at MTiOpenScreen: 7173 stereoisomer drugs (4574 single isomer molecules)	25 min	http://fafdrugs4.mti.univ-paris-diderot.fr/mobyle.html	19
2	**MTiOpenScreen** Screening Drugs-lib with Vina docking against 3 cancer protein targets	-protein MOL2 file prepared with Chimera -Drugs-lib library containing 7173 stereoisomer drugs	Top ranked 1500 ligands (with 3 best scored poses per ligand)	1 h 30 per protein	http://bioserv.rpbs.univ-paris-diderot.fr/services/MTiOpenScreen/	20
3A	**AMMOS2** Minimization Refinement: Full protein flexibility: Case 1	-protein PDB file prepared with Chimera# -top ranked 1000 ligand poses generated in Step 2	1000 minimized ligand poses ranked by binding energy	8 min per protein	http://drugmod.rpbs.univ-paris-diderot.fr/ammosHome.php	21
3B	**AMMOS2** Minimization Refinement: Protein flexibility: Case 3, all protein atoms within 6 Å of ligand	-protein PDB file prepared with Chimera# -4500 ligand poses generated in Step 2 (1500 top ranked ligands x 3 best poses)	4500 minimized ligand poses ranked by binding energy	45 min per protein	http://drugmod.rpbs.univ-paris-diderot.fr/ammosHome.php	21
3C	**AMMOS2** Minimization Refinement: Protein flexibility Case 4, protein sidechain atoms within 6 Å of ligand	-protein PDB file prepared with Chimera# -4500 ligand poses generated in Step 2 (1500 top ranked ligands x 3 best poses)	4500 minimized ligand poses ranked by binding energy	45 min per protein	http://drugmod.rpbs.univ-paris-diderot.fr/ammosHome.php	21

The protein receptor preparation can be achieved using a two-step protocol with the user-friendly visualization tool Chimera, which is a free to academic and nonprofit users. All details on the Chimera’s two-step protocol can be found in the Supplementary Information of Ref. 42.
